# Association Between Cumulative Exposure to Increased Low-Density Lipoprotein Cholesterol and the New Occurrence of Peripheral Artery Disease

**DOI:** 10.3389/fneur.2021.696695

**Published:** 2021-10-21

**Authors:** Xinmin Liu, Yu Wang, Jianwei Wu, Anxin Wang, Xiaoli Zhang, Zhentang Cao, Xingquan Zhao

**Affiliations:** ^1^Department of Neurology, Beijing Tiantan Hospital, Capital Medical University, Beijing, China; ^2^China National Clinical Research Center for Neurological Diseases, Beijing, China; ^3^Department of Epidemiology and Health Statistics, School of Public Health, Capital Medical University, Beijing, China; ^4^Beijing Municipal Key Laboratory of Clinical Epidemiology, Beijing, China; ^5^Research Unit of Artificial Intelligence in Cerebrovascular Disease, Chinese Academy of Medical Sciences, Beijing, China

**Keywords:** LDL-C, non-HDLC, PAD, epidemiology, risk factor

## Abstract

**Background and Purpose:** Peripheral artery disease (PAD) is a manifestation of systemic atherosclerosis with increased risk of severe cardiovascular and cerebrovascular events. The relationship between one-time measuring of low-density lipoprotein cholesterol (LDL-C) and PAD is inconsistent. Increasing evidence shows that the predictive value of non-high-density lipoprotein cholesterol (non-HDLC) on atherosclerosis disease is superior to LDL-C. We aimed to investigate the relationship between cumulative exposure to increased LDL-C and the risk of newly developed PAD and compare the predictive value of LDL-C with non-HDLC.

**Materials and Methods:** In the Asymptomatic Polyvascular Abnormalities Community study, we enrolled 2,923 participants with LDL-C and non-HDLC measured every 2 years from 2006 to 2012. Cumulative exposure to increased LDL-C and non-HDLC, defined as LDL-C burden and non-HDLC burden, respectively, was calculated as the weighted sum of the difference between the measured value and the cutoff value. A new occurrence of PAD was identified through ankle brachial index measured in 2010 and 2012. Multivariate models were adopted to analyze the association of LDL-C burden and non-HDLC burden with the newly developed PAD. The receiver operating curve was drawn, and the area under the curve was calculated to compare the predictive performance of LDL-C burden with a single measure of LDL-C in 2006 and non-HDL-C burden adjusted with a model including traditional risk factors.

**Results:** Of the 2,923 participants, 5.4% (158/2,923) were diagnosed as newly developed PAD. In the univariate analysis, the highest quartile of LDL-C burden was significantly associated with new occurrence of PAD [odds ratio (OR) 1.75, 95% confidence interval (CI) 1.13–2.73]. After adjustment for confounding factors, the same result was obtained (OR 1.59, 95%CI 1.01–2.49). The non-HDLC burden failed to show any statistical significance on the newly developed PAD (OR 1.31, 95% CI 0.84–2.04). Though LDL-C burden had a tendency to show better predictive performance than non-HDLC, it did not reach statistical significance (AUC_LDL−C_ = 0.554 *vs*. AUC_non−HDLC_ = 0.544, *P* = 0.655).

**Conclusions:** Cumulative exposure to increased LDL-C is an independent risk factor of newly developed PAD. The predictive value of non-HDLC burden was not revealed.

## Introduction

Peripheral artery disease (PAD) is an atherosclerotic occlusive disease in which plaque accumulates in the distal artery, diminishing blood circulation in lower extremity arteries. In the initial stage, PAD is asymptomatic, but in the later stage, patients will feel intermittent claudication and pain during movement (1). With the aggravation of ischemia and atherosclerosis, arterial stenosis or even occlusion will appear, resulting in amputation or even death, which is also an independent risk factor for poor prognosis of cardiovascular and cerebrovascular diseases in the future (2–5). Approximately 200 million individuals worldwide suffer from PAD, with the prevalence increasing with age (6) and with an incidence rate of about 10–25% among individuals over 55 years old (4). With the coming of aging society, PAD has become a global problem in the 21st century. Previous studies have identified that increased low-density lipoprotein cholesterol (LDL-C) was an independent risk factor for an incident of a cardiocerebrovascular disease (7, 8). Although LDL-C plays a critical role in the process of atherosclerosis, there was no consistency on the relationship between one-time measuring LDL-C and PAD (9–11). Several studies demonstrated that the predictive value on atherosclerosis disease of non-HDLC is superior to LDL-C, as non-high-density lipoprotein cholesterol (non-HDLC) comprises all atherogenic lipoproteins, including LDL-C, intermediate-density lipoprotein, very-low-density lipoprotein, and lipoprotein(a) (12, 13). Therefore, we aimed to explore the association between cumulative exposure to increased LDL-C and newly developed PAD and compare the predictive value of LDL-C with non-HDLC.

## Method

### Study Design and Population

**Figure 1:**
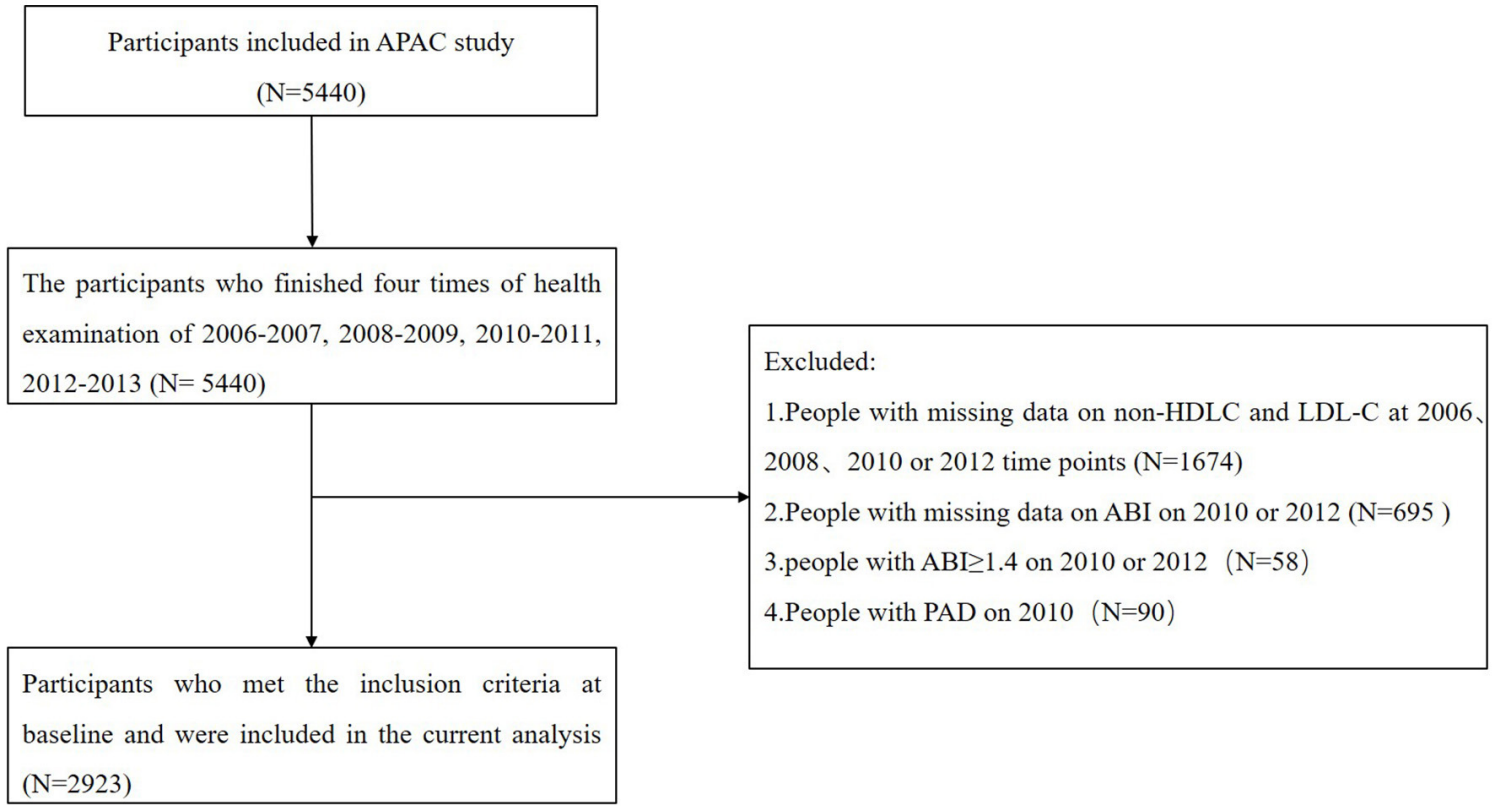
Study flow chart.

The Asymptomatic Polyvascular Abnormalities Community (APAC) study is a community-based, prospective cohort study (14). As a subset of the Kailuan study (15), the inclusion criteria were as follows: (1) age ≥40 years and (2) without cardiovascular or cerebrovascular diseases, including transient ischemic attack, stroke, and coronary disease at baseline. From June 2006 to October 2007, a total of 5,440 participants were enrolled in the cohort. They underwent four exams at 2-year intervals from 2006 to 2012. We excluded 1,674 participants with missing data of LDL-C or non-HDLC at 2006, 2008, 2010, or 2012 time points and 695 participants without the ankle brachial index (ABI) values in 2010 or 2012. Moreover, 58 other participants with elevated ABI values ≥1.40 and 90 participants diagnosed with PAD in 2010 were also excluded. Finally, 2,923 participants were included in the current study (Figure 1).

### Measurement of Lipid Levels and Calculation of Lipid Burden

Fast blood samples were taken from the antecubital vein and analyzed within 4 h using an auto-analyzer (Hitachi 747; Hitachi, Tokyo, Japan) at the laboratory research center of Kailuan Hospital. The lipid parameters, including LDL-C, high-density lipoprotein cholesterol (HDL-C), total cholesterol (TC), and triglyceride (TG), were measured at every 2-year follow-up in 2006, 2008, 2010, and 2012. The non-HDLC level was calculated as total TC minus HDL-C.

Cumulative exposure to increased LDL-C and non-HDLC is defined as LDL-C burden and non-HDLC burden, respectively. LDL-C burden was calculated as the weighted sum of the difference between the measured value and the ideal cutoff value. The cutoff value of LDL-C was set at 1.8 mmol/L, which was the target to prevent stroke for high-risk patients as recommended in the 2019 European guideline (16, 17). However, no target of non-HDLC was recommended in any guideline. The non-HDLC burden is calculated as the weighted sum of the average level:


$$\begin{array}{l} \text{LDL}-\text{C }{\text{burden}}_{2006-2008}=[(\text{LDL}-{\text{C}}_{2006}-1.8)\\ +\;(\text{LDL}-{\text{C}}_{2008}-1.8)]/2\;\times {\text{time}}_{2006-2008}\\ \text{Non}-\text{HDLC }{\text{burden}}_{2006-2008}=(\text{non}-{\text{HDLC}}_{2006}\\ +\text{ non}-{\text{HDLC}}_{2008})\text{​}/2\;\times {\text{ time}}_{2006-2008}\\ (\text{LDL}-\text{C or non}-\text{HDLC})\;{\text{burden}}_{2006-2012}=\\ (\text{LDL}-\text{C or non}-\text{HDLC})\;{\text{burden}}_{2006-2008}\;+\\ (\text{LDL}-\text{C or non}-\text{HDLC})\;{\text{burden}}_{2008-2010}\;+\\ (\text{LDL}-\text{C or non}-\text{HDLC}){\text{ burden}}_{2010-2012} \end{array}$$

The LDL-C burden and non-HDLC burden were all divided into four groups according to their quartiles.

### Assessment of PAD

The measurement of ABI was completed through a portable Doppler device (Hokanson MD6 Doppler with MD6VR Chart Recorder; Bellevue, WA, USA). After a 10-min rest in the supine position, the systolic pressure for each leg was recorded, and the ABI was calculated separately. The lower ABI value was used for the analysis. PAD was defined as ABI ≤ 0.90 (18). Besides this, ABI ≥1.40 was considered abnormal, suggesting poorly compressible leg arteries, and was excluded. The new occurrence of PAD was defined as ABI >0.90 in 2010, but ≤ 0.90 in 2012.

### Demographic and Clinical Information

Demographic information including age and sex, behavior risk factors such as smoking and drinking, physical activity, income status, medical history, and medication history were acquired through a standardized questionnaire at baseline in 2006. The systolic blood pressure (SBP) and diastolic blood pressure were measured. Body mass index (BMI) was calculated as weight (kg) divided by the square of height (m^2^). Besides this, data from laboratory tests including lipid parameters, fasting blood glucose, and uric acid were also collected.

### Statistical Analysis

Continuous variables were described by medians with interquartile ranges because of skewed distributions. Categorical variables were presented as counts and percentages. For continuous variables, the Wilcoxon test was used to perform comparisons. For categorical variables, the chi-square test was used.

The LDL-C burden and non-HDLC burden were divided into four groups according to their quartiles, and the lowest quartile was defined as the reference group. Univariate and multivariate logistic regression analyses were used to evaluate the association of LDL-C burden and non-HDLC burden with the new occurrence of PAD. In addition, we further performed a logistic regression model with restricted cubic splines for LDL-C burden and non-HDL-C burden for the new occurrence of PAD. The five knots for spline were placed at the 5, 25, 50, 75, and 95th percentiles of the LDL-C burden and non-HDL-C burden. The receiver operating curve was drawn, and the area under the curve (AUC) was calculated to compare the predictive performance of LDL-C burden with a single measure of LDL-C in 2006 and non-HDL-C burden adjusted with a model including traditional risk factors.

A two-sided *P* < 0.05 was considered statistically significant. All analyses were performed by SAS software, version 9.4 (SAS Institute Inc., Cary, NC).

## Results

### Baseline Characteristics

Of the 2,923 participants enrolled in the current analysis, 5.4% (158/2,923) were diagnosed as new occurrence of PAD. As shown in Table 1, the LDL-C levels at every follow-up time point and total LDL-C burden were significantly higher in those with new occurrence of PAD, who were also more likely to have higher SBP and BMI. The non-HDLC levels were significantly higher in 2006 and 2008. There was no significant difference in age, sex, physical activity, income status, smoking status, drinking status, medical history including diabetes mellitus, hypertension, and hypercholesterolemia, and medication history including antihypertensive medication, antidiabetic medication, and lipid-lowering medication. Additionally, no significant differences were seen for TC, TG, and HDL-C.

**Table 1:** Baseline characteristics.

	**Total patients**	**Non-PAD**	**New occurrence of PAD**	***P*** **-value**
	**(***n*** = 2,923)**	**(***n*** = 2,765)**	**(***n*** = 158)**	
Age, years	49.92 (44.53, 58.02)	49.92 (44.59, 57.72)	50.02 (43.73, 65.53)	0.47
Male, *n* (%)	1,674(57.27)	1,581 (57.18)	93 (58.86)	0.74
SBP, mmHg	121.33 (110, 138.67)	120.67 (110.50, 138.33)	130.00 (110.00, 140.00)	0.05
DBP, mmHg	80.00 (72.00, 89.00)	80.00 (72.00, 88.67)	80.00 (70.67, 90.00)	0.29
BMI, kg/m^2^	24.62 (22.52, 27.02)	24.57 (22.49, 26.90)	26.15 (23.47, 28.62)	<0.001
FBG, mmol/L	5.10 (4.63, 5.69)	5.09 (4.63, 5.66)	5.20 (4.77, 5.80)	0.093
UA, μmol/L	274.00 (223.87, 335.87)	274.00 (223.00, 335.00)	278.44 (227.00, 347.00)	0.29
TC, mmol/L	4.91 (4.28, 5.54)	4.91 (4.28, 5.52)	5.05 (4.36, 5.81)	0.09
TG, mmol/L	1.23 (0.87, 1.85)	1.23 (0.86, 1.84)	1.34 (0.93, 2.09)	0.06
HDL-C, mmol/L	1.50 (1.30, 1.75)	1.50 (1.30, 1.75)	1.45 (1.28, 1.72)	0.15
**LDL-C**				
LDL-C_2006_, mmol/L	2.30 (1.87, 2.76)	2.30 (1.87, 2.75)	2.39 (1.86, 2.92)	0.32
LDL-C_2008_, mmol/L	2.52 (1.98, 3.00)	2.51 (1.97, 3.00)	2.65 (2.05, 3.24)	0.02
LDL-C_2010_, mmol/L	2.56 (2.11, 2.97)	2.55 (2.11, 2.96)	2.71 (2.13, 3.19)	0.03
LDL-C burden_2006−2008_ (mmol/L) * year	1.22 (0.37, 2.02)	1.22 (0.36, 2.00)	1.31 (0.67, 2.58)	0.05
LDL-C burden_2008−2010_ (mmol/L) * year	1.37 (0.57, 2.25)	1.36 (0.56, 2.24)	1.53 (0.85, 2.42)	0.03
LDL-C burden (mmol/L) * year	4.59 (2.07, 6.52)	4.55 (2.02, 6.44)	5.13 (2.72, 7.60)	0.02
**Non-HDLC**				
Non-HDLC_2006_, mmol/L	3.36 (2.76, 4.00)	3.35 (2.75, 3.98)	3.50 (2.87, 4.30)	0.03
Non-HDLC_2008_, mmol/L	3.45 (2.85, 3.99)	3.45 (2.85, 3.97)	3.62 (2.86, 4.32)	0.05
Non-HDLC_2010_, mmol/L	3.29 (2.72, 3.96)	3.28 (2.73, 3.93)	1.75 (2.56, 4.25)	0.13
Non-HDLC burden_2006−2008_ (mmol/L) * year	6.66 (5.35, 8.21)	6.64 (5.36, 8.18)	7.11 (5.17, 9.12)	0.08
Non-HDLC burden_2008−2010_ (mmol/L) * year	6.52 (4.91, 8.02)	6.50 (4.90, 7.99)	6.91 (4.96, 8.41)	0.14
Non-HDLC burden (mmol/L) * year	21.38 (18.14, 25.34)	21.32 (18.14, 25.25)	22.78 (18.16, 27.36)	0.06
**Physical activity**, ***n*** **(%)**				0.24
None	443 (15.16)	412 (14.9)	31 (19.62)	
Seldom	2,108 (72.12)	2,002 (72.41)	106 (67.09)	
Always	372 (12.73)	351 (12.69)	21 (13.29)	
**Income status,** ***n*** **(%)**				0.5
<600	979 (33.49)	920 (33.27)	59 (37.34)	
600–800	1,442 (49.33)	1,373 (49.66)	69 (43.67)	
800–1,000	252 (8.62)	237 (8.57)	15 (9.49)	
>1,000	250 (8.55)	235 (8.50)	15 (9.49)	
Current smoker, *n* (%)	798 (27.30)	762 (27.56)	36 (22.78)	0.2
Current drinker, *n* (%)	1,043 (35.68)	992 (35.88)	51 (32.28)	0.39
**Medical history,** ***n*** **(%)**				
Diabetes mellitus	78 (2.67)	72 (2.60)	6 (3.80)	0.31
Hypertension	323 (11.05)	300 (10.85)	23 (14.56)	0.15
Hypercholesterolemia	212 (7.25)	199 (7.2)	13 (8.23)	0.64
Antihypertensive medication, *n* (%)	283 (9.68)	263 (9.51)	20 (12.66)	0.21
Antidiabetic medication, *n* (%)	65 (2.22)	61 (2.21)	4 (2.53)	0.78
Lipid-lowering medication, *n* (%)	29 (0.99)	27 (0.98)	2 (1.27)	0.67

*SBP, systolic blood pressure; DBP, diastolic blood pressure; BMI, body mass index; UA, uric acid; FBG, fasting blood glucose; TC, total cholesterol; TG, triglyceride; HDL-C, high-density lipoprotein cholesterol; LDL-C, low-density lipoprotein cholesterol; non-HDL-C burden, non-HDLC burden_2006−2012_; LDL-C burden, LDL-C burden_2006−2012_*.

### Association Between LDL-C Burden or Non-HDLC Burden With Newly Developed PAD

**Figure 2:**
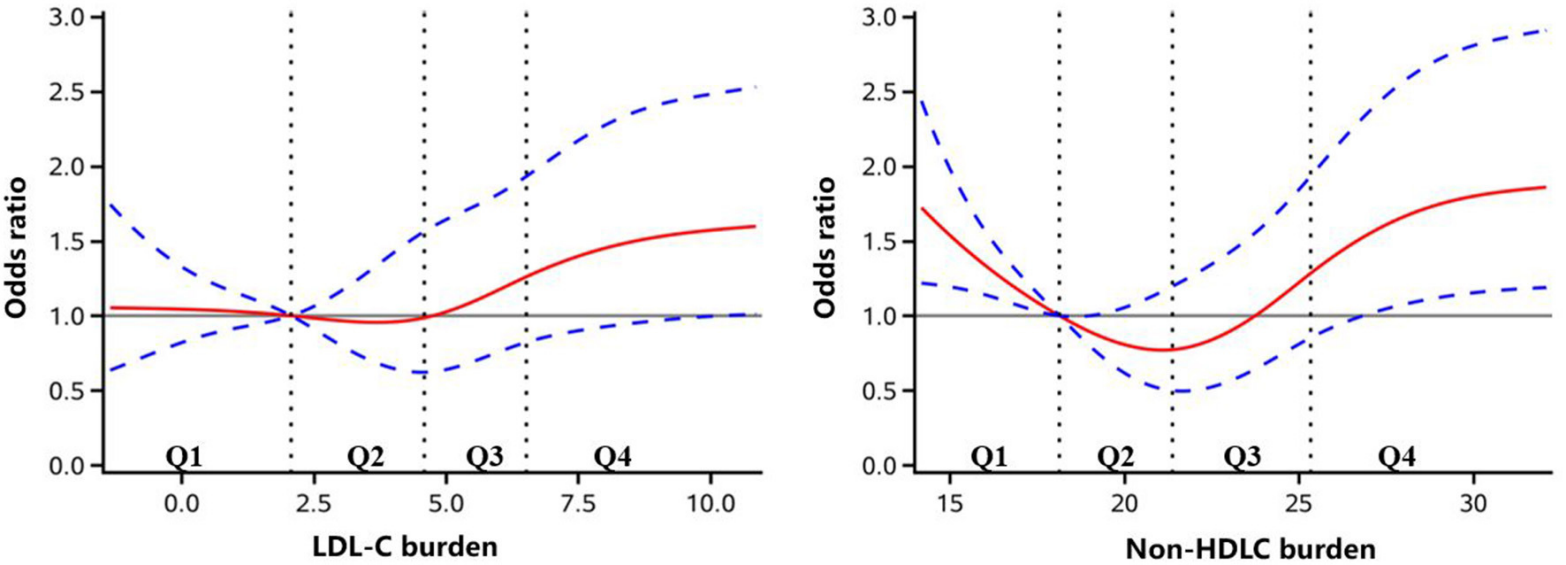
Adjusted odds ratio of newly developed peripheral artery disease according to low-density lipoprotein cholesterol (LDL-C) and non-high-density lipoprotein cholesterol (non-HDLC) burden. The red lines indicate adjusted odds ratio, and the blue dotted line indicate the 95% confidence interval bands. The reference is Q1 of LDL-C and non-HDLC burden. The data were fitted with a logistic regression model of restricted cubic spline with five knots (5, 25, 50, 75, and 95th percentiles) for LDL-C and non-HDLC burden, with adjustment for age, sex, smoker, drinker, body mass index, diabetes mellitus, hypertension, hypercholesterolemia, physical activity, and income status. The lowest 5% and highest 5% of the participants were not shown for small sample sizes.

As shown in Table 2, in a univariate analysis, compared to the lowest quartile, the highest quartile of LDL-C burden demonstrated a significant association with new occurrence of PAD (OR 1.75, 95% CI 1.13–2.73). In a multivariate analysis, after adjustment for age and sex, the same results were reached (OR 1.69, 95% CI 1.08–2.63). When further adjusting for other risk factors, including smoking status, drinking status, BMI, diabetes mellitus, hypertension, hypercholesterolemia, physical activity, and income status, the association between LDL-C burden and new occurrence of PAD remained (OR 1.59, 95% CI 1.01–2.49). However, no significant association of non-HDLC with new occurrence of PAD was obtained by multivariate analysis (Table 3). Through further logistic regression analyses with restricted cubic spline (Figure 2), we observed a U-shaped association between non-HDLC burden and new occurrence of PAD. The relationship between LDL-C burden and new occurrence of PAD tended to be S-shaped (Figure 2).

**Table 2:** Odds ratios (OR) for newly developed peripheral artery disease (PAD) for low-density lipoprotein cholesterol (LDL-C) burden.

**Newly developed PAD**						
**Variable**	**Crude**		**Model 1^a^**		**Model 2^b^**	
	**OR (95% CI)**	***P*** **-value**	**OR (95% CI)**	***P*** **-value**	**OR (95% CI)**	***P*** **-value**
**LDL-C**						
Q1	1		1		1	
Q2	1.09 (0.67, 1.78)	0.72	1.09 (0.67, 1.77)	0.72	1.05 (0.64, 1.71)	0.85
Q3	1.00 (0.61, 1.64)	1	1.00 (0.61, 1.65)	0.99	0.99 (0.60, 1.62)	0.95
Q4	1.75 (1.13, 2.73)	0.01	1.69 (1.08, 2.63)	0.02	1.59 (1.01, 2.49)	0.04

a *Adjusted for age and sex*.

b *Adjusted for model 1 plus smoking status, drinking status, body mass index, diabetes mellitus, hypertension, hypercholesterolemia, physical activity, and income status*.

**Table 3:** Odds ratios (OR) for newly developed peripheral artery disease (PAD) for non-high-density lipoprotein cholesterol (non-HDLC) burden.

**Newly developed PAD**						
**Variable**	**Crude**		**Model 1^a^**		**Model 2^b^**	
	**OR (95%CI)**	***P*** **-value**	**OR (95%CI)**	***P*** **-value**	**OR (95%CI)**	***P*** **-value**
**Non-HDLC**						
Q1	1		1		1	
Q2	0.81 (0.50, 1.31)	0.39	0.79 (0.49, 1.28)	0.34	0.75 (0.46, 1.22)	0.24
Q3	0.65 (0.39, 1.09)	0.1	0.617 (0.37, 1.03)	0.06	0.56 (0.33, 0.94)	0.03
Q4	1.61 (1.07, 2.44)	0.02	1.48 (0.97, 2.26)	0.07	1.31 (0.84, 2.04)	0.23

a *Adjusted for age and sex*.

b *Adjusted for model 1 plus smoking status, drinking status, body mass index, diabetes mellitus, hypertension, hypercholesterolemia, physical activity, and income status*.

**Figure 3:**
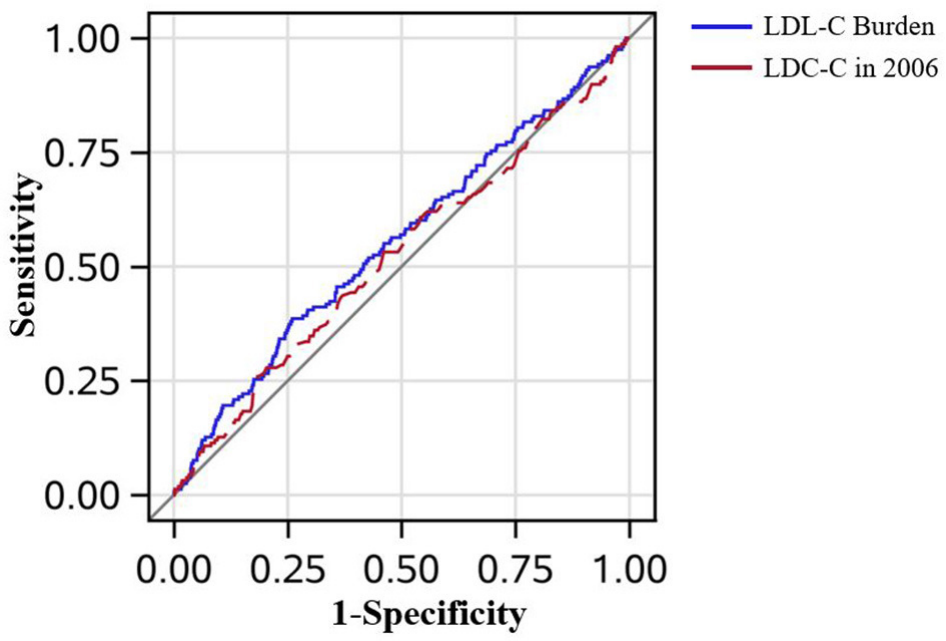
The receiver operating characteristic curves in all patients. The low-density lipoprotein cholesterol (LDL-C) burden had the tendency to perform better than a single measure of LDL-C in predicting peripheral artery disease after adjustment for age, sex, smoker, drinker, body mass index, diabetes mellitus, hypertension, hypercholesterolemia, physical activity, and income status (0.554 *vs*. 0.524, *P* = 0.160).

**Figure 4:**
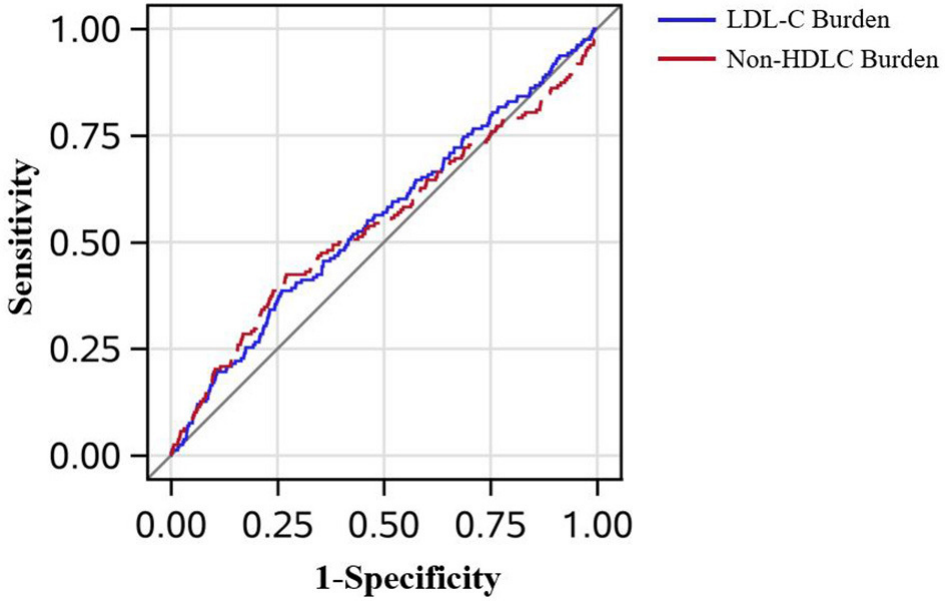
The receiver operating characteristic curves in all patients. The low-density lipoprotein cholesterol burden had the tendency to perform better than the non-high-density lipoprotein cholesterol burden in predicting peripheral artery disease after adjustment for age, sex, smoker, drinker, body mass index, diabetes mellitus, hypertension, hypercholesterolemia, physical activity, and income status (0.554 *vs*. 0.544, *P* = 0.655).

In Figure 3, the AUC of LDL-C burden and a single measure of LDL-C in 2006 adjusted by age, sex, smoker, drinker, BMI, diabetes mellitus, hypertension, hypercholesterolemia, physical activity, and income status in predicting PAD is demonstrated. Though LDL-C burden had a tendency to show better predictive performance than a single measure of LDL-C in 2006, it did not reach statistical significance (AUC_LDL−C_ = 0.554 *vs*. AUC_LDL−C2006_ = 0.524, *P* = 0.160). In Figure 4, LDL-C burden had a tendency to show better predictive performance than non-HDLC, but it did not reach statistical significance (AUC_LDL−C_ = 0.554 *vs*. AUC_non−HDLC_ = 0.544, *P* = 0.655).

## Discussion

In this large prospective cohort study, we demonstrated that cumulative exposure to increased LDL-C is significantly associated with newly developed PAD. However, the predictive value of non-HDLC burden was not revealed.

In a prospective study involving 27,888 female participants, the rate of incident symptomatic PAD was 0.39% (110/27,888), with the average follow-up period having a median of 15.1 years (9). It was similar to another large-scale prospective study, with the rate of newly developed symptomatic PAD on females at 0.36% (100/27,935) (19). In addition, another study which enrolled 14,916 males aged 40–84 years found that the incidence of new occurrence of symptomatic PAD was 0.93% (140/14,916) (10). In our study, PAD was assessed as ABI < 0.90, including the asymptomatic and symptomatic patients. The current study showed that the rate of newly incident PAD was 5.4% (158/2,923). Another large-scale study showed that the rate of ABI declined to 0.9 or less than that of the elderly cohort over 6 years which was 9.5% (218/2,289) (11). They included a population aged over 65 years old, while we included all age groups older than 40 years old, which may be the reason for the difference. Thus, the incidence of asymptomatic PAD in the population is high, which is not found and valued due to the lack of early symptoms. Therefore, it is fundamental to pay attention to the risk factors of the formation and aggravation of PAD.

Previous studies focusing on the relationship of LDL-C with PAD have reached an inconsistent conclusion. Aday et al. (9) conducted a prospective study with follow-up for a median of 15.1 years and found no significant association of LDL-C and new occurrence of PAD. Additionally, Pradhan et al. (19) yielded the same results. However, Ridker et al. (10). found that LDL-C was an independent risk factor for incident symptomatic PAD in a prospective cohort with an average follow-up period of 9 years. Kennedy et al. (11) found that LDL-C was an independent predictor of ABI decline in the elderly cohort over 6 years. In our study, LDL-C burden had a tendency to show better predictive performance than a single measure of LDL-C in 2006, but it did not reach statistical significance. As we know, the level of LDL-C is fluctuating by the short-time diet and physical exercise of people. The LDL-C burden in our study calculated by the weighted sum of the average is a constant and reliable index to reflect long-term LDL-C levels. Although the better prediction performance of LDL-C burden was not seen at present, the prediction value of burden could be more significant with the extension of follow-up time. We found that the highest quartile of LDL-C burden was significantly associated with new occurrence of PAD, which provided strong evidence that increased LDL-C was an independent risk factor of new onset of PAD. However, in restricted cubic spline, the second quartile was of lower risk than the first quartile, which did not reach statistical significance. The reason could be some important covariates, such as obesity burden, which were not included. Furthermore, the patients diagnosed with a new occurrence of PAD were only 158. It is warranted to be investigated in a larger population.

However, we did not find a significant association between non-HDLC burden and new occurrence of PAD. Previous studies have not reached a unified conclusion (9, 19, 20). Several studies found that non-HDLC was a better predictor of cardiovascular and cerebrovascular diseases than LDL-C (12, 13). However, in our study, non-HDLC burden failed to show a higher predictive value than LDL-C burden, similar to a previous APAC study (21). Only two large-scale cohort studies on the risk factors of PAD studied the relationship between LDL-C and non-HDLC in newly onset symptomatic PAD, respectively (9, 19). Unfortunately, the association of neither LDL-C nor non-HDLC with PAD reached statistical significance. Thus, it was ambiguous whether the predictive value of non-HDLC burden was better than LDL-C burden on the new occurrence of PAD. What is more, there was no recommended cutoff value for non-HDLC. In our study, non-HDLC burden was calculated as the weighted sum of the average level, which may not be as meaningful as the increased LDL-C level. We observed a U-shaped association between non-HDLC burden and new occurrence of PAD. The first quartile was of higher risk than the second and third quartiles. When the TG levels are over 2.3 mmol/L, non-HDLC is an optimal index to represent all atherogenic cholesterol (22). In the current analysis, the median (interquartile range) of TG level was 1.23 (0.87–1.85) mmol/L, which could be the reason why the superiority of non-HDLC burden on predictive value was not revealed. HDL-C is regarded as “good cholesterol.” However, this view has been challenged in recent years. The concentration of HDL-C does not fully represent its dynamic transmission capacity. Some trials trying to improve cardiovascular outcomes by increasing HDL-C have not been successful (23). Studies also found that the incidence rate and mortality of atherosclerosis could increase in individuals with a very high level of HDL-C (24). Non-HDLC level was calculated as total TC minus HDL-C. Therefore, a U-shaped association between non-HDLC burden and new occurrence of PAD was observed.

To our best knowledge, this study was the first large cohort study to investigate the association of LDL-C burden and new occurrence of PAD. However, there are still several limitations in our study. First, we defined PAD through ABI and excluded participants with ABI ≥1.4, which might result in an underestimated incidence of PAD. Second, our study population was based on a randomly selected subgroup of participants of the Kailuan study. It may not be representative of the population of the Tangshan area in Hebei province despite the large study sample. Our study could have bias due to the population used. Last but not least, ABI was only measured at a single time point in 2010 and 2012. No baseline ABI value was measured in 2006.

In conclusion, we found that cumulative exposure to increased LDL-C was an independent risk factor of newly developed PAD. The predictive value of non-HDLC burden was not revealed.

## Data Availability Statement

The raw data supporting the conclusions of this article will be made available by the authors, without undue reservation.

## Ethics Statement

This study was conducted in accordance with the guidelines from the Helsinki Declaration and approved by the Ethics Committees of the Kailuan General Hospital (Approval No: 2006-05) and Beijing Tiantan Hospital (Approval No: 2010-014-01). Written informed consent was obtained from all participants.

## Author Contributions

XL and YW interpreted the data and drafted the manuscript. XZhao conceived and designed the research. AW and XZhan acquired and analyzed the data. ZC made critical revision of the manuscript. All authors approved of the revision and agreed to be accountable for the content of work.

## Funding

The APAC study was supported by grants from Chinese Academy of Medical Sciences Innovation Fund for Medical Sciences (2019-I2M-5-029), Beijing Municipal Committee of Science and Technology (Z201100005620010), National Key Research and Development Program of China (2018YFC1312204), and Beijing Natural Science Foundation (Z200016).

## Conflict of Interest

The authors declare that the research was conducted in the absence of any commercial or financial relationships that could be construed as a potential conflict of interest.

## Publisher's Note

All claims expressed in this article are solely those of the authors and do not necessarily represent those of their affiliated organizations, or those of the publisher, the editors and the reviewers. Any product that may be evaluated in this article, or claim that may be made by its manufacturer, is not guaranteed or endorsed by the publisher.

## Abbreviations

PAD: peripheral artery disease
LDL-C: low-density lipoprotein cholesterol
non-HDLC: non-high-density lipoprotein cholesterol
TC: total cholesterol
TG: triglyceride
HDL-C: high-density lipoprotein cholesterol
SBP: systolic blood pressure
DBP: diastolic blood pressure
BMI: body mass index
UA: uric acid
FBG: fasting blood glucose
ROC: receiver operating curve
AUC: area under the curve.
